# Design of Secure Protocol for Cloud-Assisted Electronic Health Record System Using Blockchain

**DOI:** 10.3390/s20102913

**Published:** 2020-05-21

**Authors:** MyeongHyun Kim, SungJin Yu, JoonYoung Lee, YoHan Park, YoungHo Park

**Affiliations:** 1 School of Electronics Engineering, Kyungpook National University, Daegu 41566, Korea; kimmyeong123@knu.ac.kr (M.K.); harry250@knu.ac.kr (J.L.); 2 School of Computer Engineering, Keimyung University, Daegu 42601, Korea; yhpark@kmu.ac.kr

**Keywords:** security protocol, cloud, blockchain, electronic health record, BAN logic, AVISPA simulation

## Abstract

In the traditional electronic health record (EHR) management system, each medical service center manages their own health records, respectively, which are difficult to share on the different medical platforms. Recently, blockchain technology is one of the popular alternatives to enable medical service centers based on different platforms to share EHRs. However, it is hard to store whole EHR data in blockchain because of the size and the price of blockchain. To resolve this problem, cloud computing is considered as a promising solution. Cloud computing offers advantageous properties such as storage availability and scalability. Unfortunately, the EHR system with cloud computing can be vulnerable to various attacks because the sensitive data is sent over a public channel. We propose the secure protocol for cloud-assisted EHR system using blockchain. In the proposed scheme, blockchain technology is used to provide data integrity and access control using log transactions and the cloud server stores and manages the patient’s EHRs to provide secure storage resources. We use an elliptic curve cryptosystems (ECC) to provide secure health data sharing with cloud computing. We demonstrate that the proposed EHR system can prevent various attacks by using informal security analysis and automated validation of internet security protocols and applications (AVISPA) simulation. Furthermore, we prove that the proposed EHR system provides secure mutual authentication using BAN logic analysis. We then compare the computation overhead, communication overhead, and security properties with existing schemes. Consequently, the proposed EHR system is suitable for the practical healthcare system considering security and efficiency.

## 1. Introduction

As patient healthcare records have been developed from traditional paper management to electronic record management, they can be safely stored and accessed and authorized only by legitimate medical centers [1]. With the electronic health record (EHR) management system, storage availability and historical errors can be minimized, improving the availability and accuracy of healthcare records. EHR systems can help people to prevent diseases and enhance the cure rate, and ensures great convenience for medical centers and patients. However, health-related information from each healthcare system is stored in their own medical servers, respectively, in traditional EHR systems [2]. Therefore, when the patients transfer from a hospital to another one, hospitals should establish a point-to-point channel to share patients information. Furthermore, the traditional EHR system is generally established as a centralized system so that it has a single point of failure. Blockchain can serve as a helpful method to solve these problems.

In the last few years, numerous blockchain-based EHR system studies have been presented to address the problems of traditional EHR system and improve efficiency [3,4,5]. Blockchain is a network technology that ensures the decentralization and integrity of information by sharing records with multiple distributed nodes [6,7]. Blockchain is considered as a trusted distributed ledger that keeps transactions in a chain of chronological blocks linked through hash values. In addition, the blockchain has properties such as data anonymity, decentralization, and so on. In particular, many blockchain studies have presented various models such as ethereum and hyperledger [8]. Although both models have similar structures, hyperledger is relatively better in terms of network performance and energy efficiency [9]. Furthermore, hyperledger fabric [10] aims to solve the bottleneck problem of a cloud system and enables users to keep ownership of their own data, as well as to share data securely with feedback. However, the EHR system should consider that it is hard to store whole EHR data in blockchain because of the size and the price of blockchain [11]. Thus, if there is a sudden and unexpected demand for storage and resources, blockchain-based EHR systems should guarantee sufficient capacities.

In the last few years, many blockchain-based EHR systems have adopted cloud computing to enlarge scalability and to solve the storage problem associated with blockchain [12,13]. As an important technology to improve the development of smart medical services, cloud technology can serve as a platform for sharing information between remote hospitals and can solve the problem of remote collaboration diagnostic [14,15]. The health information can be efficiently managed on a cloud server facilitating precise and accurate diagnosis and treatment, as well as the development of various healthcare services [16]. Unfortunately, the cloud-based EHR system can be vulnerable to potential attacks because the sensitive data is sent over a public channel. To resolve these security problems, the cloud-based EHR systems require a secure and efficient protocol. Thus, we develop the security protocol using elliptic curve cryptosystems (ECC) that provides high security level, and efficient computation and communication overheads even in small storage spaces.

Recently, numerous EHR systems have been presented that combine blockchain, cloud, and authentication to solve each problem associated with cloud and blockchain [17,18]. Kaur et al. [17] presented a model architecture for EHR data using blockchain in the cloud environment to provide secure healthcare services. Furthermore, Nagasubramanian et al. [18] presented a cloud-assisted secure E-health record system using blockchain to provide integrity and decentralization for the EHR sharing and health diagnosis. However, these cloud-assisted EHR systems using blockchain [17,18] do not specifically address a secure protocol for registration, authentication, transaction uploading, and so on. Therefore, we propose the secure protocol for cloud-assisted EHR system using blockchain to guarantee security, integrity, and decentralization for EHR sharing and health diagnosis. The proposed EHR system utilizes the cloud technology to achieve storage efficiency, and the data in each block only stores metadata to increase block construction efficiency and minimize distributed storage waste. Furthermore, in the proposed EHR system, blockchain technology is used to efficiently provide data integrity and access control using log transactions. Moreover, the proposed EHR system provides secure health data sharing in a public channel using ECC.

### 1.1. Research Contributions

The detailed contributions in this paper are summarized as below.

- We propose the secure protocol for cloud-assisted EHR system using blockchain. The proposed scheme combines cloud computing, blockchain, and authentication to provide a secure and effective medical diagnosis for legitimate patients.
- The proposed scheme withstands various attacks, including impersonation, session key disclosure, and replay attacks, and also provides secure mutual authentication and anonymity.
- We present the Burrows–Abadi–Needham (BAN) logic analysis [19,20] to analyze that the proposed scheme provides secure mutual authentication.
- We perform the automated validation of internet security protocols and applications (AVISPA) [21,22] to analyze against man-in-the-middle (MITM) and replay attacks. Furthermore, we show the performance analysis of the proposed scheme with existing schemes.

### 1.2. Organization

The remainder of this paper is organized as follows. Section 2 presents the related works, and Section 3 shows the preliminaries for help explanation of this paper. In Section 4 and Section 5, we introduce the system model and also propose a secure protocol for cloud-assisted EHR system using blockchain. Section 6 performs the security analysis of the proposed scheme using informal and formal security analysis. In Section 7, we compare the performance analysis of the proposed scheme with related schemes. Finally, we summarize the paper in Section 8.

## 2. Related Works

In the past decades, many authentication schemes in the healthcare system have been presented to ensure secure healthcare service and EHR sharing [23,24,25,26]. Kumar et al. [23] presented an efficient authentication scheme for healthcare applications in wireless medical sensor networks to provide secure healthcare services. Wu et al. [24] presented a reliable RFID-based authentication scheme in healthcare environments. Their scheme [24] does not reveal any private data, including the identity number and the health data of the legitimate patient. Liu et al. [25] presented a remote authentication protocol for wireless body area networks. Their scheme [25] is not suitable for limited-resource wearable sensor devices because it utilizes bilinear pairing cryptography with high computation and communication overheads. Renuka et al. [26] presented a three-factor authentication protocol for smart healthcare using ECC. Renuka et al. [26] demonstrated that their scheme can prevent against various attacks. However, their schemes for the healthcare system [23,24,25,26] are essentially a centralized system so that these schemes do not solve problems such as the single point of failure. Therefore, a blockchain mechanism with decentralized properties is essential for solving the problems of centralized systems.

In the last few years, many EHR system studies have been presented using blockchain to ensure data integrity along with decentralized properties [27,28,29]. Pandey and Litoriya [27] presented secure e-health networks from counterfeit medicine penetration using blockchain. Their scheme [27] ensures data integrity and security capability properties against drug data to provide secure healthcare services. Agbo and Mahmoud [28] presented a comparison of blockchain frameworks for healthcare applications. Tanwar et al. [29] presented a blockchain-based EHR system for secure medical data sharing. Their scheme [29] can avoid the reliability problem of the trusted third parties, and also can provide secure medical services between each entity. However, these schemes for the EHR systems using blockchain [27,28,29] should consider that it is hard to store whole EHR data in blockchain because of the size and price of blockchain [11]. Therefore, if there is a sudden and unexpected demand for storage and resources, the EHR systems using blockchain have to guarantee sufficient capacities. Therefore, these schemes require a cloud-based mechanism in the EHR system to provide cloud storage technology and decentralized properties using blockchain.

Recently, numerous cloud-based EHR system studies using the blockchain have been presented to solve the storage overload problem associated with blockchain [30,31,32]. Wang et al. [30] presented a cloud-assisted EHR sharing to ensure security and privacy using blockchain. Their scheme [30] uses searchable encryption and proxy re-encryption to realize data security and access control. Chen et al. [31] designed a secure storage scheme based on blockchain and cloud storage to manage personal health data. Cheng et al. [32] presented a secure medical data sharing scheme based on blockchain utilizing cloud techniques. Their scheme [32] uses bilinear mapping to provide secure medical data sharing and low storage and computing overhead. However, these cloud-based EHR systems using blockchain [30,31,32] have been studied so far, but a secure authentication scheme for EHR sharing has not been specifically considered. Therefore, we present a secure cloud-assisted EHR system using blockchain to ensure secure EHR sharing.

## 3. Preliminaries

In this section, we introduce the preliminaries for help explanation of this paper.

### 3.1. Adversary Model

We present the widely used Dolev–Yao (DY) model [33] to analyze the security of the proposed protocol. The detailed assumptions of the DY model are as follows.

- An attacker can delete, inject, eavesdrop, and intercept the messages transmitted over a public channel.
- An attacker can steal the smartcard of legitimate patients and can extract secret values stored in a smartcard using power-analysis [34,35].
- An attacker may attempt various attacks such as impersonation, MITM, replay, session key disclosure attacks, and so on [36,37].

### 3.2. Hyperledger Fabric

In 2015, hyperledger fabric [10] was presented as an open source blockchain proposed by the Linux Foundation. The goal of this technology is to promote cross-industry cooperation using blockchain. Hyperledger fabric does not require digital currency and provides various advantages such as blockchain performance and reliability. Hyperledger fabric uses practical byzantine fault-tolerant (PBFT) consensus algorithm [38,39]. Therefore, we apply the PBFT algorithm to the proposed system to provide an effective consensus ability. The hyperledger architecture consists of six blockchain components:

1. Membership Service Provider (MSP): MSP is a component that validates and authenticates credentials and defines the rules for accessing a network. The MSP manages user identities and authenticates all participants in the network, making hyperledger fabric available as both private and permissioned networks. This includes providing credentials for the clients to propose transactions. As a result, a single hyperledger fabric network can be controlled by multiple MSPs.
2. Smart Contract: The smart contract of hyperledger fabric is called chaincode. Chaincode is software that defines assets and related transactions. The chaincode is called when the application needs to interact with the ledger. Every chaincode has an attached endorsement policy, which applies to all smart contracts defined in it. This identifies the organizations that need to sign transactions generated by smart contracts. In addition, smart contracts have the advantage of being able to make different smart contracts within the channel or across different channels.
3. Ordering Service: The ordering service packages a transaction in blocks and delivers it to the channel’s peers. It ensures the transaction delivery via the network. It communicates with peers and endorsing peers.
4. Identity: Each node in the network peer, client, ordered, and the manager has a digital identity with the format of certificate X.509. This identity is used to verify at every stage of the transaction to ensure if the source of the transaction is a valid source. In addition to multiple assurances, validation, and version control checks that occur, there are ongoing identity verifications happening during each stage of the transaction flow.
5. Channels: Hyperledger fabric networks can have multiple channels. Channels allow organizations to use the same network while maintaining separation between multiple blockchain. Only the peer of the channel can provide to see transactions made by all members of the channel.
6. Peer Nodes: Peer nodes constitute a fundamental element of the network as they host smart contracts within the ledger. Peer nodes execute chaincode, access ledger data, approve transactions, and interface with applications.

## 4. Cloud-Assisted EHR System Model Using Blockchain

**Figure 1:**
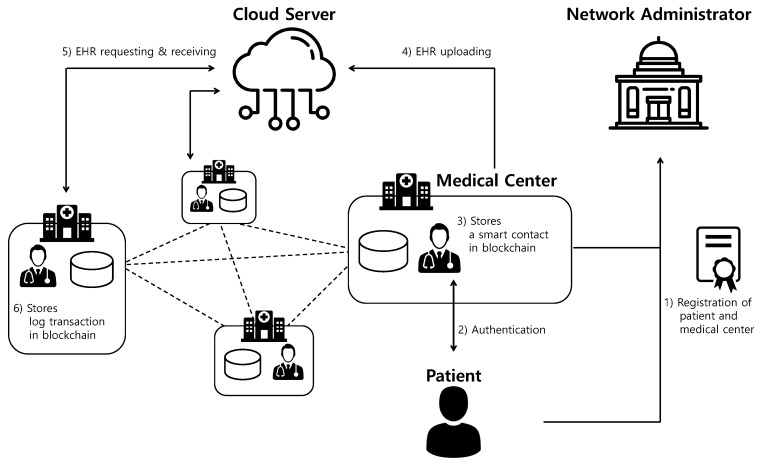
Proposed cloud-assisted electronic health record (EHR) system model using blockchain.

We introduce a cloud-assisted EHR system based on a hyperledger fabric in Figure 1. To improve the security and efficiency of medical data, this system is built on medical centers that share EHR in specific regions. The system model for the EHR comprises the four entities: the patient, the medical center, the cloud server, and the network administrator. The detailed descriptions of each entity are described as follows.

**1.**  Patient: A patient transmits the health data to the medical center in order to receive healthcare services through healthcare devices and wearable sensors. Health data of the patient are recorded in EHR with healthcare services provided by the medical center.
**2.**  Medical Centers: The medical centers are registered by the network administrator and participate in the private blockchain. The medical centers generate EHR and store it to the cloud server for sharing with other medical centers. When the medical centers view the EHRs of other medical center’s the patient, they upload a log of EHR data to the blockchain as a transaction form.
**3.**  Network Administrator: A network administrator is a trusted entity, responsible for the registration of participants, that manages the private blockchain.
**4.**  Cloud Server: A cloud server is a trusted entity that has sufficient computing power and capacity. The cloud server stores and manages the patient’s EHRs to provide secure data sharing and storage resources. A cloud server receives the EHR data from the medical center and sends the EHR to other medical centers requesting the EHR using a pre-shared secret key.

The communication flows of the proposed EHR system are described as follows.

**1.**  Patient and doctor register their identities with the help of a network administrator to access EHR services.
**2.**  Patient and doctor authenticate each other and establish a session key for future secure communication.
**3.**  The medical center receives the information for a smart contract from the patient using a session key. Then, the medical center generates a patient’s smart contract and EHR. After that, the medical center uploads a smart contract at the blockchain.
**4.**  The medical center encrypts EHRs of the legitimate patient using a pre-shared secret key and sends it to the cloud server. Then, the cloud server decrypts the encrypted EHR data and stores EHR data in the database.
**5.**  The other medical center requests the EHR data of the medical center to the cloud server. Next, the cloud server encrypts EHR data of the medical center using a pre-shared secret key and sends it to the other medical center.
**6.**  Finally, the medical center decrypts the encrypted EHR data and then uploads the log transaction, including the patient and medical center masked identities, signatures, and timestamps at the blockchain.

## 5. Proposed Protocol for Cloud-Assisted EHR System Using Blockchain

We present a secure protocol for cloud-assisted EHR system using hyperledger fabric. The proposed EHR system is that only the EHRs can be outsourced by authenticated participants and each operation on outsourcing EHRs is integrated into the blockchain as a transaction. The proposed scheme consists of six phases: the registration, authentication, smart contract uploading, EHR storing, EHR requesting, and log transaction uploading. Before the registration phase, a network administrator (NA) sets up the networks. The NA selects a base point *G* over an elliptic curve $E_{p}$ with order *p* that is a large prime number. *P* of order *q* is one of *G*’s generators, in which *q* is a large prime number. Then, the NA selects a secret key $s_{NA}$ and generates a public key $PK_{NA}=s_{NA}\cdot G$. Finally, NA shares the network configuration and policies with all system participants. Furthermore, the NA publishes, $\left\{p,q,G,P,PK_{NA}\right\}$ as system parameters, and a cloud server (CS) establishes a secure pre-shared key with medical centers. Table 1 illustrates the notations used in the proposed scheme.

### 5.1. Registration Phase

In the proposed scheme, the registration phase consists of the patient registration and the medical center registration.

#### 5.1.1. Patient Registration Phase

**Figure 2:**
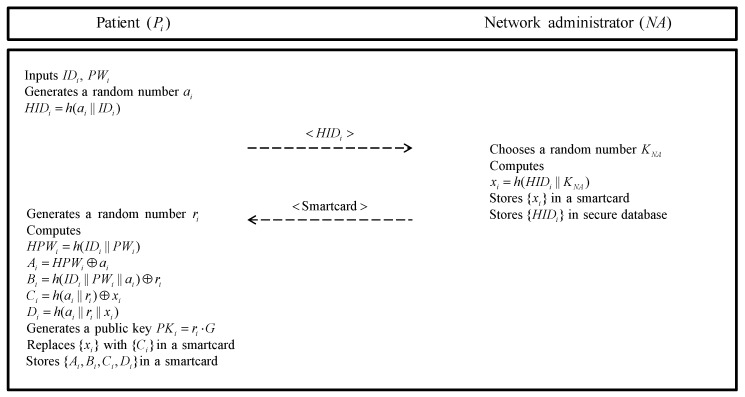
Patient registration phase of the proposed protocol.

If a patient ($P_{i}$) wants to receive a medical diagnosis, the $P_{i}$ must first register his/her information with the NA and generate a private key and a public key. The patient registration phase is executed over a secure channel. Figure 2 shows the patient registration phase and detailed steps are as follows.

**Step 1:**  The $P_{i}$ requests registration to the network administrator NA. First, $P_{i}$ inputs identity $ID_{i}$ and password $PW_{i}$. Then, the $P_{i}$ generates a random number $a_{i}$ and computes $HID_{i}=h(a_{i}||ID_{i})$ and sends $HID_{i}$ to the NA.
**Step 2:**  The NA chooses a random number $K_{NA}$ and computes $x_{i}=h(HID_{i}\left|\right|K_{NA})$ using the $HID_{i}$ received from the $P_{i}$. Then, the NA stores $\left\{x_{i}\right\}$ into the smartcard and issues it to the $P_{i}$ in the blockchain. Finally, the NA stores $\left\{HID_{i}\right\}$ in secure database.
**Step 3:**  After the $P_{i}$ receives smartcard from the NA, the $P_{i}$ generates a random number $r_{i}$ as a secret key. $P_{i}$ computes $HPW_{i}=h(ID_{i}||PW_{i})$, $A_{i}=HPW_{i}⊕a_{i}$, $B_{i}=h(ID_{i}||PW_{i}\left|\right|a_{i})⊕r_{i}$, $C_{i}\;=\;h(a_{i}\left|\right|r_{i})⊕x_{i}$ and $D_{i}=h(a_{i}\left|\right|r_{i}\left|\right|x_{i})$. And then, the $P_{i}$ generates a public key $PK_{i}=r_{i}\cdot G$ and replaces $\left\{x_{i}\right\}$ with $\left\{C_{i}\right\}$ in a smartcard. Finally, $P_{i}$ stores $\left\{A_{i},B_{i},C_{i},D_{i}\right\}$ in the smartcard.

#### 5.1.2. Medical Center Registration Phase

**Figure 3:**
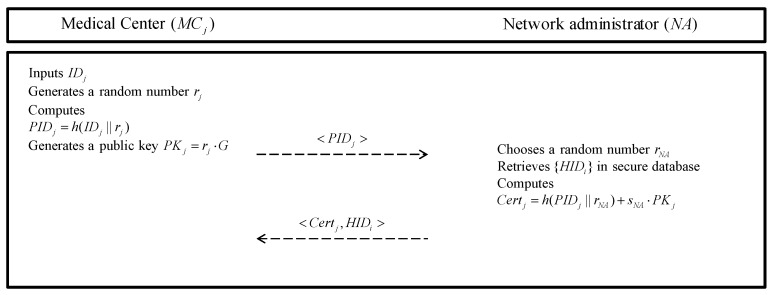
Medical center registration phase of the proposed protocol.

A medical center ($MC_{j}$) must register with the NA to have a key agreement with patients and exchange information with other related medical centers. The masked identity of the $MC_{j}$ is shared with other entities. This registration phase is also executed over a secure channel. The detailed steps are described as follows and are illustrated in Figure 3.

**Step 1:**  A medical center $MC_{j}$ chooses a unique identity $ID_{j}$ and generates a random number $r_{j}$ as a its secret key. Then, the $MC_{j}$ computes a masked identity $PID_{j}=h(ID_{j}\left|\right|r_{j})$ and generates a public key $PK_{j}=r_{j}\cdot G$. $MC_{j}$ sends $PID_{j}$ to the NA.
**Step 2:**  After receiving registration request message, the NA chooses a random number $r_{NA}$ and retrieves $\left\{HID_{i}\right\}$ in secure database. Then, the NA computes $Cert_{j}=h(PID_{j}\left|\right|r_{NA})+s_{NA}\cdot PK_{j}$. The NA stores $Cert_{j}$ with $PID_{j}$ and sends $\left\{Cert_{i},HID_{i}\right\}$ to $MC_{j}$.
**Step 3:**  After the $MC_{j}$ receives the messages, the $MC_{j}$ stores $\left\{Cert_{j},HID_{i}\right\}$ in secure database.

### 5.2. Authentication Phase

**Figure 4:**
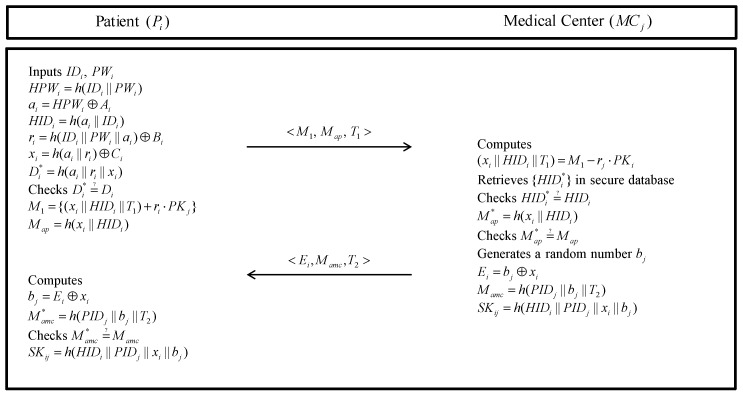
Authentication phase of the proposed protocol.

If the $P_{i}$ wants a secure health diagnosis, the patient and medical center must establish a session key. The detailed steps are as following in Figure 4.

**Step 1:**  The $P_{i}$ inputs his/her $ID_{i}$, $PW_{i}$, and smartcard. Then, the smartcard computes $HPW_{i}=h(ID_{i}||PW_{i})$, $a_{i}=HPW_{i}⊕A_{i}$, $HID_{i}=h(a_{i}||ID_{i})$, $r_{i}=h(ID_{i}||PW_{i}\left|\right|a_{i})⊕B_{i}$, $x_{i}=h(a_{i}\left|\right|r_{i})⊕C_{i}$, and $D_{i}^{*}=h(a_{i}\left|\right|r_{i}\left|\right|x_{i})$. Then, the smartcard checks whether $D_{i}^{*}\overset{?}{=}D_{i}$. If it is correct, the $P_{i}$ generates a timestamp $T_{1}$ and encrypts messages $M_{1}=\{(x_{i}||HID_{i}\left|\right|T_{1})+r_{i}\cdot PK_{j}\}$ and computes $M_{ap}=h(x_{i}||HID_{i})$. Next, the $P_{i}$ sends a message $<M_{1},M_{ap},T_{1}>$ to $MC_{j}$ via a public channel.
**Step 2:**  After receiving the message $<M_{1},M_{ap},T_{1}>$, the $MC_{j}$ decrypts $(x_{i}||HID_{i}\left|\right|T_{1})=M_{1}-r_{j}\cdot PK_{i}$. After that, the $MC_{j}$ retrieves $HID_{i}^{*}$ in secure database and checks whether $HID_{i}^{*}\overset{?}{=}HID_{i}$. If it is correct, the $MC_{j}$ computes $M_{ap}^{*}=h(x_{i}||HID_{i})$ and checks whether $M_{ap}^{*}\overset{?}{=}M_{ap}$. If it is valid, the $MC_{j}$ generates a random number $b_{j}$ and timestamp $T_{2}$ and calculates $E_{i}=b_{j}⊕x_{i}$, $M_{amc}=h(PID_{j}\left|\right|b_{j}\left|\right|T_{2})$. $HID_{i}$ updates at the proper period. After that, the $MC_{j}$ generates a session key $SK_{ij}=h(HID_{i}||PID_{j}\left|\right|x_{i}\left|\right|b_{j})$. Finally, $MC_{j}$ sends message $<E_{i},M_{amc},T_{2}>$ to $P_{i}$ over an open channel.
**Step 3:**  When the $P_{i}$ receives the message from the $MC_{j}$, the $P_{i}$ computes $b_{j}=E_{i}⊕x_{i}$, and $M_{amc}^{*}=h(PID_{j}\left|\right|b_{j}\left|\right|T_{2})$. Then, the $P_{i}$ checks whether $M_{amc}^{*}\overset{?}{=}M_{amc}$. If it is valid, the $P_{i}$ computes a session key $SK_{ij}=h(HID_{i}||PID_{j}\left|\right|x_{i}\left|\right|b_{j})$.

### 5.3. Smart Contract Uploading Phase

**Figure 5:**
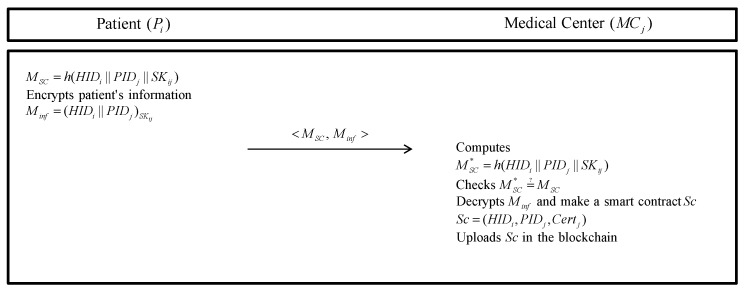
Smart contract uploading phase of the proposed protocol.

After receiving information for the smart contract from the $P_{i}$, the $MC_{j}$ generates a smart contract and then uploads the smart contract in the blockchain. The detailed steps are as following in Figure 5.

**Step 1:**  The $P_{i}$ generates message $M_{sc}=h(HID_{i}||PID_{j}||SK_{ij})$ and encrypts his/her information with $SK_{ij}$; $M_{inf}=(HID_{i}||PID_{j}{)}_{SK_{ij}}$. Then, the $P_{i}$ sends $<M_{sc},M_{inf}>$ to the $MC_{j}$.
**Step 2:**  The $MC_{j}$ computes $M_{sc}^{*}=h(HID_{i}||PID_{j}||SK_{ij})$ and checks $M_{SC}^{*}\overset{?}{=}M_{SC}$. If it is valid, $MC_{j}$ decrypts $M_{inf}$ and generates a smart contract Sc using $\left(HID_{i},PID_{j},Cert_{j}\right)$. Finally, the $MC_{j}$ uploads Sc in the blockchain.

### 5.4. EHR Storing Phase

**Figure 6:**
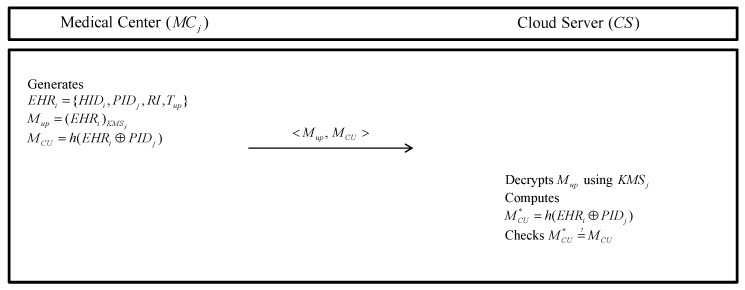
EHR storing phase of the proposed protocol.

After uploading smart contract, the $MC_{j}$ generates $EHR_{i}$ and stores $EHR_{i}$ in CS. Detailed steps are as follows in Figure 6.

**Step 1:**  The $MC_{j}$ generates $EHR_{i}$ including $HID_{i}$, $PID_{j}$, an information of health record RI, and EHR’s uploading time $T_{up}$. Then, the $MC_{j}$ encrypts $EHR_{i}$ using a secure pre-shared key $M_{up}={\left(EHR_{i}\right)}_{KMS_{j}}$ and computes $M_{CU}=h\left(EHR_{i}⊕PID_{j}\right)$. Finally, the $MC_{j}$ sends $<M_{up},M_{CU}>$ to the CS.
**Step 2:**  The CS decrypts $M_{up}$ with $KMS_{j}$, computes $M_{CU}^{*}=h\left(EHR_{i}⊕PID_{j}\right)$ and checks $M_{CU}^{*}\overset{?}{=}M_{CU}$. If it is correct, the CS stores $EHR_{i}$ in the server database.

### 5.5. EHR Requesting Phase

**Figure 7:**
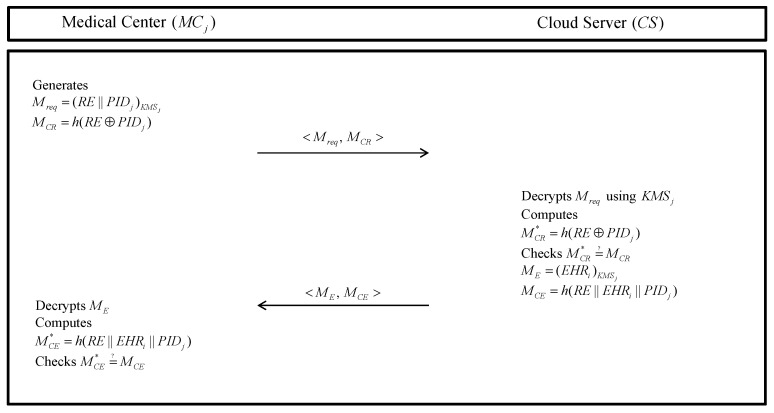
EHR requesting phase of the proposed protocol.

If the $MC_{j}$ wants to confirm $EHR_{i}$, $MC_{j}$ sends request messages to the CS. Then, the CS sends $EHR_{i}$ to $MC_{j}$. Detailed steps are as follows in Figure 7.

**Step 1:**  The $MC_{j}$ generates request messages RE and encrypts $M_{req}=(RE||PID_{j}{)}_{KMS_{j}}$ using $KMS_{j}$ and computes $M_{CR}=h\left(RE⊕PID_{j}\right)$. Then, the $MC_{j}$ sends $<M_{req},M_{CR}>$ to the CS.
**Step 2:**  After receiving the messages $<M_{req},M_{CR}>$, the CS decrypts $M_{req}$ with $KMS_{j}$. After that, the CS computes $M_{CR}^{*}=h\left(RE⊕PID_{j}\right)$ and checks $M_{CR}^{*}\overset{?}{=}M_{CR}$. If it is correct, the CS retrieves $EHR_{i}$ corresponding request. The CS encrypts $EHR_{i}$ with $KMS_{j}$ and calculates $M_{CE}=h(RE||EHR_{i}||PID_{j})$. After then, the CS sends $<M_{E},M_{CE}>$ to the $MC_{j}$.
**Step 3:**  $MC_{j}$ decrypts the received $M_{E}$ with $KMS_{j}$ and computes $M_{CE}^{*}=h(RE||EHR_{i}||PID_{j})$. Then, the $MC_{j}$ checks $M_{CE}^{*}\overset{?}{=}M_{CE}$. If it is not valid, the $MC_{j}$ eliminates communication and received data.

### 5.6. Log Transaction Uploading Phase

**Figure 8:**
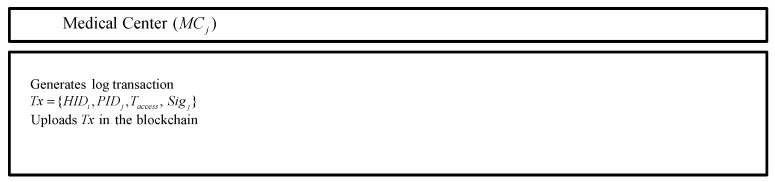
Log transaction uploading phase of the proposed protocol.

After $MC_{j}$ receives $EHR_{i}$ from CS, $MC_{j}$ generates a log transaction and uploads the log transaction in the blockchain. The $MC_{j}$ generates a log transaction $Tx=\{HID_{i},PID_{j},T_{access},Sig_{j}\}$, where $T_{access}$ is accessing time of $EHR_{i}$ and $Sig_{j}$ is a signature of the $MC_{j}$. Finally, the $MC_{j}$ uploads Tx in the blockchain. The detailed step is as following in Figure 8.

## 6. Security Analysis

In this section, we analyze the proposed protocol as a security aspect. We show that the proposed protocol is secure against malicious attacks using informal analysis. We also prove that the proposed protocol can provide secure mutual authentication using a widely adopted BAN logic. In addition, we simulate Automated Validation of Internet Security Protocols and Applications (AVISPA) to prove that the proposed protocol is secure against MITM and replay attacks.

### 6.1. Informal Security Analysis

We analyze the proposed protocol to perform informal security analysis and show the protocol can resist various attacks. Moreover, we show that our protocol can provide secure mutual authentication and patient’s anonymity.

#### 6.1.1. Impersonation Attack

A malicious adversary $M_{A}$ tries to impersonate a legitimate patient $P_{i}$ to obtain sensitive information. To impersonate $P_{i}$, the $M_{A}$ has to successfully compute a message $<M_{1},M_{ap},T_{1}>$. However, the $M_{ap}$ is masked with a secret value $x_{i}$ and the adversary cannot compute $x_{i}$ because he/she does not know a random number $K_{NA}$. Moreover, the $M_{1}$ is encrypted by the $P_{i}$’s secret key. Therefore, the proposed protocol is secure against impersonation attacks.

#### 6.1.2. Session Key Disclosure Attack

If the $M_{A}$ wants to generate a legitimate session key $SK_{ij}=h(HID_{i}||PID_{j}\left|\right|x_{i}\left|\right|b_{j})$, the $M_{A}$ must know random number $b_{j}$. However, the $M_{A}$ cannot obtain $b_{j}$. Moreover, the $M_{A}$ cannot reveal real the identities of $P_{i}$ and $MC_{j}$ because they are masked with random numbers $a_{i}$ and $r_{j}$. Therefore, the proposed protocol can prevent session key disclosure attacks.

#### 6.1.3. Perfect Forward Secrecy

Even if a $M_{A}$ knows a long-term private secret key $s_{NA}$, the $M_{A}$ cannot obtain the previous session key, because a session key $SK_{ij}=h(HID_{i}||PID_{j}\left|\right|x_{i}\left|\right|b_{j})$ does not include $s_{NA}$. Further, if the long-term private parameter $K_{NA}$ is compromised, the $M_{A}$ cannot obtain $x_{i}$. Because $x_{i}$ is masked with $HID_{i}$ and $HID_{i}$ is masked with a random number $a_{i}$. Therefore, the proposed protocol guarantees perfect forward secrecy.

#### 6.1.4. Replay Attack

Suppose a $M_{A}$ learns transmitted messages performing a replay attack. However, the $M_{A}$ cannot use previous messages, because transmitted messages include timestamps, and $P_{i}$ and $MC_{j}$ check the timestamps are correct. Then, they check that $M_{ap}^{*}\overset{?}{=}M_{ap}$ and $M_{amc}^{*}\overset{?}{=}M_{amc}$ are correct. Thus, the proposed protocol can resist replay attacks.

#### 6.1.5. Privileged Insider Attack

Suppose a privileged insider user of the system, the user is an insider adversary. The insider adversary knows the registration information $<HID_{i}>$ of a legitimate user. Moreover, the adversary also can know stored values $\left\{A_{i},B_{i},C_{i},D_{i}\right\}$ in the smartcard to perform power analysis attacks. However, stored values in the smartcard are masked with $HPW_{i}$. Therefore, the adversary cannot know $HPW_{i}$ that cannot guess a valid password. Therefore, the proposed protocol prevents privileged insider attack.

#### 6.1.6. Anonymity

A $M_{A}$ cannot reveal a legitimate patient’s real identity $ID_{i}$, because $ID_{i}$ is masked by hash function or encryption with random numbers or secret key. Therefore, our protocol provides the patient’s anonymity.

#### 6.1.7. Mutual Authentication

According to Section 6.1.1, the $M_{A}$ cannot compute a valid session key and cannot impersonate a legitimate patient. Moreover, $P_{i}$ and $MC_{j}$ check a legitimate entity to verify whether $M_{ap}^{*}\overset{?}{=}M_{ap}$ and $M_{amc}^{*}\overset{?}{=}M_{amc}$ are correct. If the conditions are correct, the $P_{i}$ and $MC_{j}$ authenticate each other. Therefore, our protocol can provide secure mutual authentication.

### 6.2. BAN Logic Analysis

We demonstrate that the proposed protocol provides secure mutual authentication between *P* and MC using BAN logic [19,20]. Table 2 presents BAN logic notations. In addition, we define the rules, goals, idealized forms, and assumptions for performing BAN logic analysis.

#### 6.2.1. BAN Logic Rules

The BAN logic rules are defined as follows.

**1.**  Message meaning rule:
  $$\frac{X\;|\equiv X\overset{K}{↔}Y,\;X⊲{\left\{Q\right\}}_{K}}{X\;\left|\equiv Y\;\right|∼Q}$$
**2.**  Nonce verification rule:
  $$\frac{X\;\left|\equiv \#(Q),\;X\;\right|\equiv Y\;|∼Q}{X\;\left|\equiv Y\;\right|\equiv Q}$$
**3.**  Jurisdiction rule:
  $$\frac{X\;\left|\equiv Y\;\right|⟹Q,\;X\;\left|\equiv Y\;\right|\equiv Q}{X\;|\equiv Q}$$
**4.**  Freshness rule:
  $$\frac{X\;|\equiv \#(Q)}{X\;|\equiv \#\left(Q,Z\right)}$$
**5.**  Belief rule:
  $$\frac{X\;|\equiv \left(Q,Z\right)}{X\;|\equiv Q}$$

#### 6.2.2. Goals

We define the security goals to prove that the proposed system is capable of performing secure mutual authentication.

**Goal 1:**  $P|\equiv (P\overset{SK}{⟷}MC)$
**Goal 2:**  $P|\equiv MC|\equiv (P\overset{SK}{⟷}MC)$
**Goal 3:**  $MC|\equiv (P\overset{SK}{⟷}MC)$
**Goal 4:**  $MC|\equiv P|\equiv (P\overset{SK}{⟷}MC)$

#### 6.2.3. Idealized Forms

We define the idealized forms as below.

$Msg_{1}$: P→MC: $\left(x_{i},HID_{i},T_{1}\right)\overset{PK_{j}}{{}_{⟶MC}}$
$Msg_{2}$: MC→P: ${\left(PID_{j},b_{j},T_{2}\right)}_{x_{i}}$

#### 6.2.4. Assumptions

The initial assumptions are given below.

$A_{1}$: $P|\equiv (P\overset{x_{i}}{⟷}MC)$
$A_{2}$: $MC|\equiv \#(PK_{j})$
$A_{3}$: $P|\equiv \#(b_{1})$
$A_{4}$: $P|\equiv MC\Rightarrow (P\overset{SK}{⟷}MC)$
$A_{5}$: $MC|\equiv P\Rightarrow (P\overset{SK}{⟷}MC)$
$A_{6}$: $MC|\equiv \#(x_{i})$
$A_{7}$: $MC|\equiv \#(T_{1})$
$A_{8}$: $P|\equiv \#(T_{2})$

#### 6.2.5. Proof Using BAN Logic

We perform the BAN logic analysis. The detailed steps are as follows.

**Step 1:**  From $Msg_{1}$ we can get,
  $$S_{1}:MC⊲\left(x_{i},HID_{i},T_{1}\right)\overset{PK_{j}}{{}_{⟶MC}}$$
**Step 2:**  From the message meaning rule with $S_{1}$ and $A_{2}$,
  $$S_{2}:MC|\equiv P|∼\left(x_{i},HID_{i},T_{1}\right)$$
**Step 3:**  We use the freshness rule with $S_{2}$ and $A_{6}$,
  $$S_{3}:MC|\equiv \#\left(x_{i},HID_{i},T_{1}\right)$$
**Step 4:**  Using the nonce verification rule with $S_{2}$ and $S_{3}$,
  $$S_{4}:MC|\equiv P|\equiv \left(x_{i},HID_{i},T_{1}\right)$$
**Step 5:**  By the Belief rule with $S_{4}$ and $A_{7}$,
  $$S_{5}:MC|\equiv P|\equiv \left(x_{i},HID_{i}\right)$$
**Step 6:**  Because of the session key $SK=h(HID_{i}||PID_{j}||x_{i}||b_{j})$, from $S_{5}$ and $A_{3}$,
  $$S_{6}:MC|\equiv P|\equiv \left(P\overset{SK}{⟷}MC\right)\;\;\left(\mathrm{Goal}4\right)$$
**Step 7:**  Using the jurisdiction rule with $S_{6}$ and $A_{5}$,
  $$S_{7}:MC|\equiv \left(P\overset{SK}{⟷}MC\right)\;\;\left(\mathrm{Goal}3\right)$$
**Step 8:**  From $Msg_{2}$ we can get,
  $$S_{8}:P⊲{\left(PID_{j},b_{j},T_{2}\right)}_{x_{i}}$$
**Step 9:**  From the message meaning rule with $S_{8}$ and $A_{1}$,
  $$S_{9}:P|\equiv MC|∼{\left(PID_{j},b_{j},T_{2}\right)}_{x_{i}}$$
**Step 10:**  We use the freshness rule with $S_{9}$ and $A_{3}$,
  $$S_{10}:P|\equiv \#{\left(PID_{j},b_{j},T_{2}\right)}_{x_{i}}$$
**Step 11:**  Using the nonce verification rule with $S_{8}$ and $S_{9}$,
  $$S_{11}:P|\equiv MC|\equiv {\left(PID_{j},b_{j},T_{2}\right)}_{x_{i}}$$
**Step 12:**  By the belief rule with $S_{11}$ and $A_{8}$,
  $$S_{12}:P|\equiv MC|\equiv {\left(PID_{j},b_{j}\right)}_{x_{i}}$$
**Step 13:**  Because of the session key $SK=h(HID_{i}||PID_{j}||x_{i}||b_{j})$, from $S_{12}$ and $A_{6}$,
  $$S_{13}:P|\equiv MC|\equiv \left(P\overset{SK}{⟷}MC\right)\;\;\left(\mathrm{Goal}2\right)$$
**Step 14:**  Using the jurisdiction rule with $S_{13}$ and $A_{4}$,
  $$S_{14}:P|\equiv \left(P\overset{SK}{⟷}MC\right)\;\;\left(\mathrm{Goal}1\right)$$

Therefore, the goals 1–4 clearly show that the proposed protocol provides secure mutual authentication between $P_{i}$ and $MC_{j}$.

### 6.3. AVISPA Analysis

**Figure 9:**
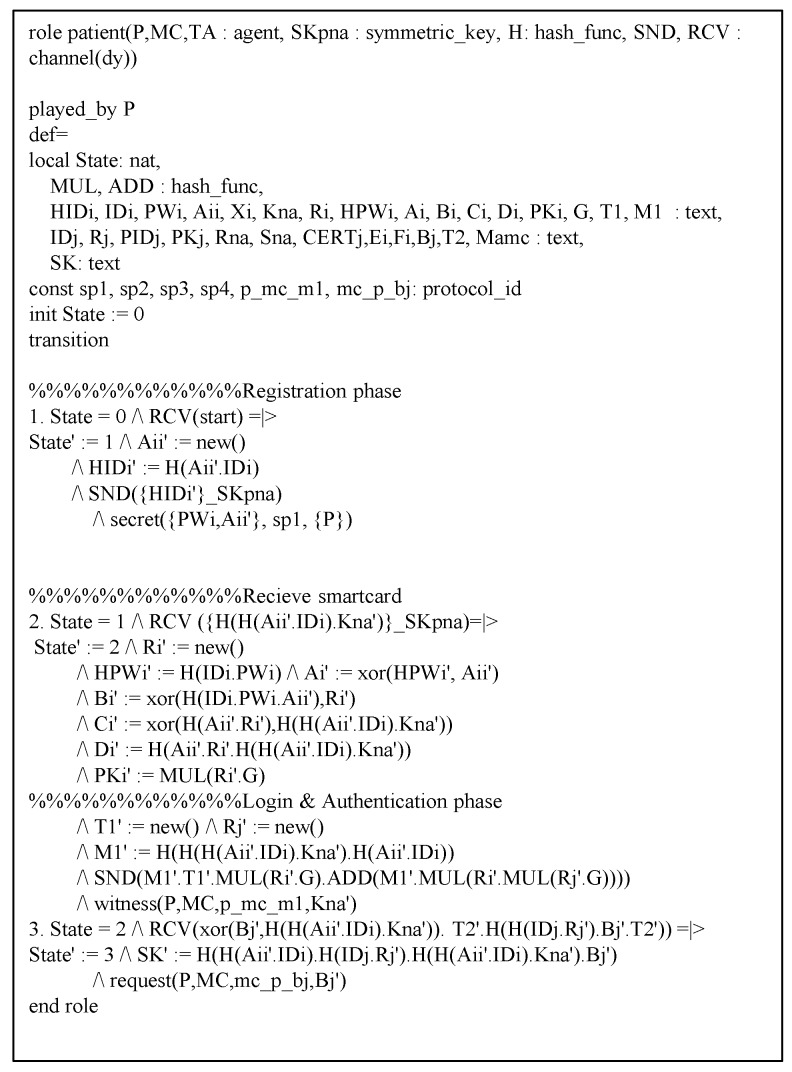
High-Level Protocol Specification Language (HLPSL) syntax of patient.

**Figure 10:**
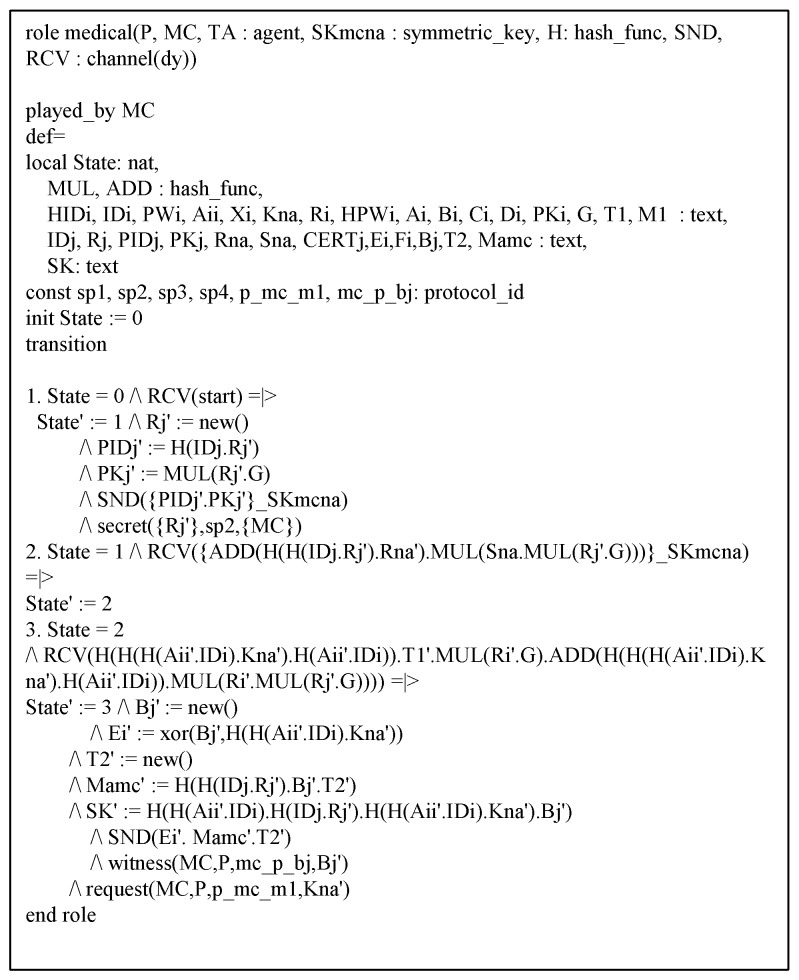
HLPSL syntax of medical center.

**Figure 11:**
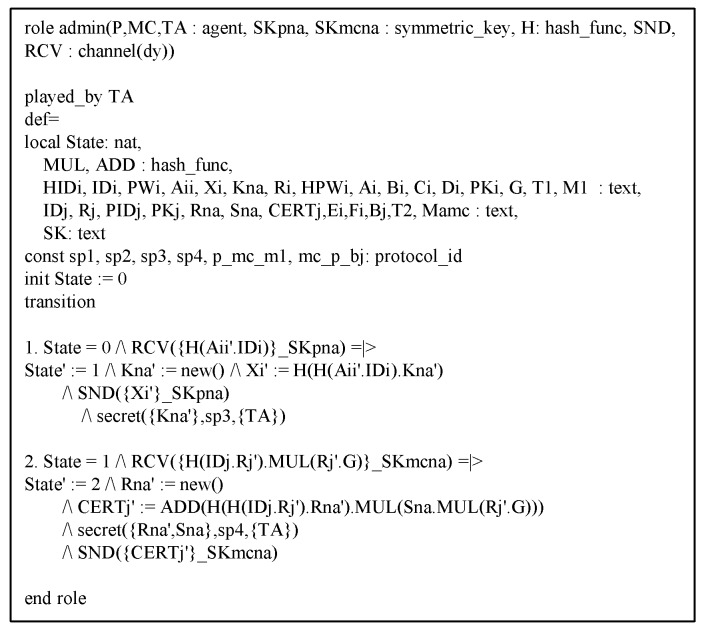
HLPSL syntax of network administrator.

**Figure 12:**
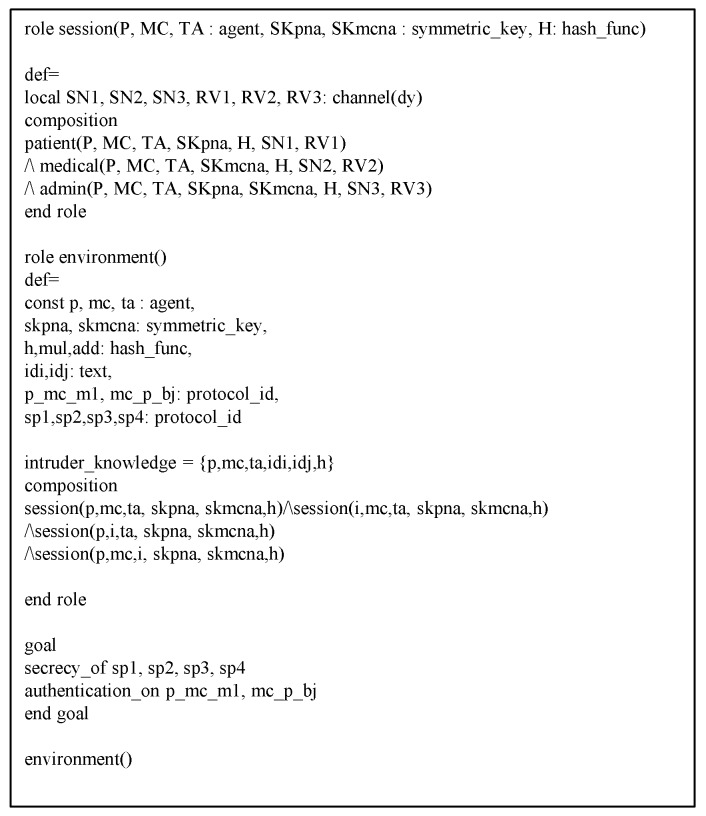
HLPSL syntax of session and environment.

**Figure 13:**
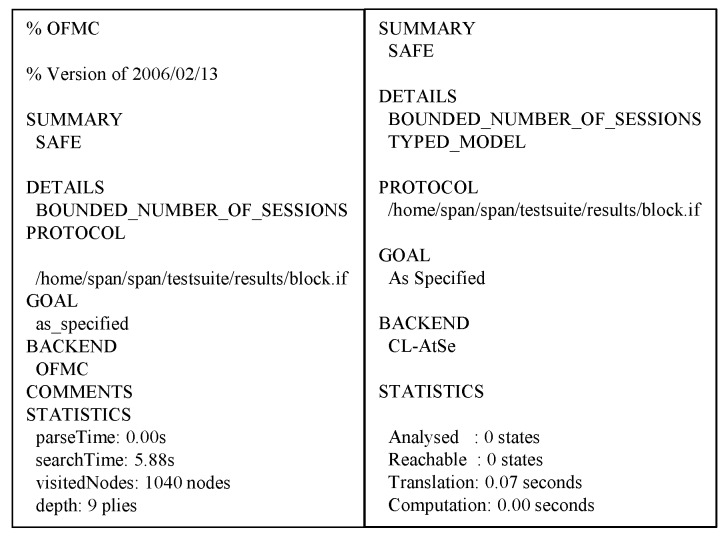
Automated Validation of Internet Security Protocols and Applications (AVISPA) analysis result using OFMC and CL-AtSe.

This section shows the proposed protocol can resist against adversary’s replay and MITM attacks to perform AVISPA simulation [21,22]. The AVISPA tool consists of High-Level Protocol Specification Language (HLPSL) [40] to generate input format (IF) of four back-ends, i.e., “On-the-Fly Model Checker (OFMC)”, “Constraint Logic-based Attack Searcher (CL-AtSe)”, “Tree automata based on Automatic Approximations for Analysis of Security Protocol (TA4SP)”, and “SAT-based Model Checker (SATMC)”. Then, the output format (OF) is created and the safety of the protocol is verified using OF. Generally, verification is performed with OFMC and CL-AtSe. The HLPSL syntax of each entity is shown in Figure 9, Figure 10 and Figure 11. Furthermore, the goal and environment of the protocol are shown in Figure 12. Goal and environment describe participants, security goals, and environment conditions. As a Figure 13, the results of AVISPA simulation under OFMC and CL-AtSe is safe. The results show that OFMC has 5.88 search time and visits 1040 nodes with 9 piles depths. Furthermore, the CL-AtSe analyzed in 0.07 seconds. Therefore, our proposed protocol provides security against MITM and replay attacks.

## 7. Performance Analysis

In this section, we analyze the computation and communication costs of the proposed protocol compared with related schemes [25,26].

### 7.1. Computation Cost

Referring to the work in [41,42,43], we compare computation costs during authentication phase for the proposed system with related schemes [25,26].

- $T_{bp}:$ The computation time of a bilinear pairing operation ≈ 4.211 ms.
- $T_{bp-sm}:$ The computation time of a scalar multiplication operation on bilinear pairing ≈1.709 ms.
- $T_{bp-ad}:$ The computation time of a point addition operation on bilinear pairing ≈0.0071 ms.
- $T_{ec-sm}:$ The computation time of a scalar multiplication operation on elliptic curve cryptography ≈0.442 ms.
- $T_{ec-ad}:$ The computation time of a point addition operation on elliptic curve cryptography ≈0.0018 ms.
- $T_{ec-enc}:$ The computation time of a encryption with elliptic curve cryptography ≈0.5102 ms.
- $T_{ec-dec}:$ The computation time of a decryption with elliptic curve cryptography ≈0.7399 ms.
- $T_{h}:$ The computation time of a one-way hash function operation ≈0.0001 ms.
- $T_{exp}:$ The computation time of an exponentiation operation ≈3.886 ms.

Table 3 shows computation costs of the proposed scheme with related schemes [25,26]. In Liu et al.’s scheme [25], a client computes $\left\{T=tP,T^{‘}=tQ_{AP}\right\}$ with multiplication on bilinear pairing, $\left\{I^{{}^{\prime }}\right\}$ with addition on bilinear pairing, {r} with exponential function, $\left\{U=kS_{2}-vs_{1}Q_{2}\right\}$ with two multiplication and one addition on bilinear pairing, and $\left\{v,key,MAC_{key}\left(v\right)\right\}$ with hash function. Then, a application provider computes {T} with multiplication on bilinear pairing, {I} with addition on bilinear pairing, $\left\{v,key,MAC_{key}\left(v\right)\right\}$ with hash function, {r} with one bilinear pairing operation, one multiplication on bilinear pairing, and one exponential function.

In Renuka et al.’s scheme [26], a user computes $\left\{V_{i},A_{i},F_{i},sk\right\}$ with two hash functions, $\left\{R_{i},E_{i},E_{s}\right\}$ with multiplication on ECC, $\left\{D_{i},H_{i}\right\}$ with one hash function. Moreover, in the registration phase, a server computes $H(B_{i})$ and stores it in memory. After that, in authentication phase, the server extracts the $H(B_{i})$. Thus, we do not include $H(B_{i})$ in the operation. Then, server computes $\{ID_{i},h\left(x⊕ID_{i}\right),h(C_{i}\left|\right|T_{1}\left|\right|E_{i}||H\left(B_{i}\right)),sk,H_{i}\}$ with one hash function, $\left\{E_{i},R_{s},E_{s}\right\}$ with multiplication on ECC.

In the proposed scheme, a patient computes $\left\{HPW_{i},r_{i},x_{i},D_{i}^{*},M_{ap},M_{amc}^{*},SK_{ij}\right\}$ with hash function, $M_{1}$ with ECC encryption. Moreover, the medical center computes $\left\{M_{1}-r_{j}\cdot PK_{i}\right\}$ with ECC decryption, $\left\{M_{ap}^{*},M_{amc},SK_{ij}\right\}$ with hash function. As a result, we provide better efficiency than existing schemes [25,26] because our scheme uses only hash function and ECC encryption/decryption.

### 7.2. Communication Cost

We compare communication costs during authentication phase for the proposed system with related schemes [25,26]. We assume that the ECC-based encryption ($EN_{ecc}$), timestamp (*T*), identity (*I*) hash function (*H*), and message authentication code (MAC) are 320, 32, 128, 160, and 160 bits [44,45], respectively. We also define that additive groups on super singular ($G_{1}$), and additive group (*G*) are 1024 and 320 bits [44,45], respectively. Table 4 shows communication costs of the proposed scheme with related schemes [25,26].

In Liu et al.’s scheme [25], transmitted messages $\left\{v,U,t_{c},T^{{}^{\prime }},I^{{}^{\prime }}\right\}$ and {MAC}. $U,T^{{}^{\prime }}$, and $I^{{}^{\prime }}$ are elements of $G_{1}$. Moreover, *v* is the element of hash function, $t_{c}$ is a timestamp, and MAC is the element of message authentication code. In Liu et al.’s scheme, transmitted messages require (160 + 1024 + 32 + 1024 + 1024 = 3264 bits) and (160 bits), respectively.

In Renuka et al.’s scheme [26], transmitted messages $\left\{D_{i},R_{i},F_{i},T_{1}\right\}$ and $\left\{R_{s},H_{i},T_{2}\right\}$. $R_{i}$ and $R_{s}$ are elements of *G*. $D_{i}$, $F_{i}$, and $H_{i}$ are elements of hash function. And also, $T_{1}$ and $T_{2}$ are elements of timestamp. In Renuka et al.’s scheme, transmitted messages require (160 + 320 + 160 + 32 = 672 bits) and (320 + 160 + 32 = 512 bits), respectively.

In the proposed scheme, transmitted messages $\left\{M_{1},M_{op},T_{1}\right\}$ and $\left\{E_{i},M_{acm},T_{2}\right\}$. $M_{op}$, $M_{acm}$, and $E_{i}$ are the elements of hash function and $M_{1}$ is the element of ECC-based encryption. And also, $T_{1}$ and $T_{2}$ are the elements of timestamp. In proposed scheme, transmitted messages require (320 + 160 + 32 = 512 bits) and (160 + 160 + 32 = 352 bits), respectively. Consequently, we provide better efficiency than related schemes [25,26] because our scheme uses hash function, timestamp, and ECC-based encryption/decryption.

### 7.3. Security Properties

Table 5 shows the comparison between the security properties of the proposed scheme and related schemes [25,26]. Our scheme guarantees perfect forward secrecy, anonymity, and mutual authentication, and avoids the single point of failure and bottleneck. In addition, the proposed scheme has the resistance of impersonation, session key disclosure, replay, and privileged insider attacks.

## 8. Conclusions

With the rapid development of the EHR system, medical centers obtain patient’s health records to provide accurate medical services through medical wearable sensors. However, these health records contain sensitive information of patients, it is necessary to ensure the security from leakage or counterfeiting in the process of storing and sharing information. Furthermore, traditional protocols for the EHR system cannot prevent the single point of failure, and the EHR system should consider storage overload problems because of the large amounts of EHR data and scalability of the system. In this paper, we proposed the secure protocol for cloud-assisted EHR system using blockchain to resolve these problems. The proposed scheme presented detailed phases for six phases such as registration, authentication, smart contract uploading, EHR storing, EHR requesting, and log transaction uploading. We proved that the proposed scheme prevents various attacks and provides secure mutual authentication, anonymity, and perfect forward secrecy. We demonstrated the safety of the proposed scheme against MITM and replay attacks using AVISPA simulation. Furthermore, we proved that the proposed scheme ensures a secure mutual authentication between patient and medical server using BAN logic. We compared the security features and performance of the proposed scheme with some existing schemes. We proved that our scheme provides better safety and efficiency than related schemes. Therefore, the proposed EHR system can be suitable for the practical healthcare system for EHRs because it is more secure and efficient than other related schemes. In the future, we aim to develop a set of realistic simulations to test the protocol. If these practical simulations are available, it will help to develop a secure protocol for the cloud-assisted EHR system using blockchain.

## Figures and Tables

**Table 1:** Notations.

Notations	Meanings
$P_{i}$	*i*-th patient
$MC_{j}$	*j*-th medical center
NA	Network Administrator
$ID_{i},ID_{j}$	Identity of $P_{i}$ and $MC_{j}$
$PW_{i}$	Password of $P_{i}$
$r_{i},r_{j}$	Secret keys of $P_{i}$ and $MC_{j}$
$s_{NA}$	Secret key of NA
$T_{1},T_{2}$	Timestamps
$T_{up},T_{access}$	Uploading/accessing time of EHR
$K_{NA},r_{NA}$	Random numbers generated by NA
$PK_{i},PK_{j}$	Public keys of $P_{i}$ and $MC_{j}$
$Cert_{i},Cert_{j}$	Certificates of $P_{i}$ and $MC_{j}$
$E_{p}\left(a,b\right)$	A nonsingular elliptic curve $y^{2}=x^{3}+ax+b$ (mod *p*)
*G*	A base point for elliptic curve
$HID_{i},PID_{j}$	Pseudo-identities of $P_{i}$ and $MC_{j}$
$T_{x}$	Log transaction
$KMS_{j}$	Secure pre-shared key among $MC_{j}$ and CS
EHR	Electronic health record
RI	Information of health record
RE	Request message of EHR
SK	Common session key shared among $P_{i}$ and $MC_{j}$
h(*)	Collision resistant one-way hash function
⊕	XOR operation
||	Concatenation operation

**Table 2:** Notations of Burrows–Abadi–Needham (BAN) logic.

Notation	Description
X|≡Q	*X***believes** statement *Q*
X|∼Q	*X* once **said** *Q*
X⇒Q	*X***controls** statement *Q*
#Q	Statement *Q* is **fresh**
X⊲Q	*X***sees** statement *Q*
$<Q{>}_{Z}$	Formula *Q* is **combined** with formula *Z*
${\left\{Q\right\}}_{K}$	*Q* is **encrypted** under key *K*
$\overset{K}{{}_{⟶Y}}$	*Y* has *K* as a **public key**
$X\overset{K}{↔}Y$	*X* and *Y* may use **shared key** *K* to communicate
SK	Session key used in the current session

**Table 3:** Computation costs of the proposed scheme with related schemes.

	Liu et al. [25]	Renuka et al. [26]	Proposed
Patient/Client	$4T_{bp-sm}+2T_{bp-ad}+T_{exp}+3T_{h}\approx 10.8643$ ms	$3T_{ec-sm}+10T_{h}\approx 1.327$ ms	$T_{ec-enc}+7T_{h}\approx 0.5109$ ms
Medical center	$2T_{bp-sm}+T_{bp-ad}+T_{exp}+T_{bp}+3T_{h}\approx 11.5863$ ms	$3T_{ec-sm}+5T_{h}\approx 1.3265$ ms	$T_{ec-dec}+3T_{h}\approx 0.7402$ ms

**Table 4:** Communication costs of the proposed scheme with related schemes.

	Liu et al. [25]	Renuka et al. [26]	Proposed
Patient/Client	$H+3G_{1}+T=3264$ bits	2H+G+T=672 bits	$EN_{ecc}+H+T=512$ bits
Medical center	MAC=160 bits	G+H+T=512 bits	2H+T=352 bits

**Table 5:** Security properties of the proposed scheme with related schemes.

	Liu et al. [25]	Renuka et al. [26]	Proposed
Impersonation attack	X	O	O
Session key disclosure attack	X	O	O
Perfect forward secrecy	X	O	O
Replay attack	O	O	O
Privileged insider attack	X	O	O
Single point of failure	X	X	O
Anonymity	O	O	O
Mutual authentication	X	O	O
Bottleneck	X	X	O
